# Weak and Saturable Protein–Surfactant Interactions in the Denaturation of Apo-α-Lactalbumin by Acidic and Lactonic Sophorolipid

**DOI:** 10.3389/fmicb.2016.01711

**Published:** 2016-11-08

**Authors:** Kell K. Andersen, Brian S. Vad, Sophie Roelants, Inge N. A. van Bogaert, Daniel E. Otzen

**Affiliations:** ^1^Interdisciplinary Nanoscience Center, Department of Molecular Biology and Genetics, Aarhus UniversityAarhus, Denmark; ^2^Agro Business Park A/STjele, Denmark; ^3^Centre for Industrial Biotechnology and Biocatalysis – InBio.be, Faculty of Bioscience Engineering, Ghent UniversityGhent, Belgium

**Keywords:** sophorolipid, acidic form, lactonic form, protein denaturation, unfolding kinetics, binding stoichiometry

## Abstract

Biosurfactants are of growing interest as sustainable alternatives to fossil-fuel-derived chemical surfactants, particularly for the detergent industry. To realize this potential, it is necessary to understand how they affect proteins which they may encounter in their applications. However, knowledge of such interactions is limited. Here, we present a study of the interactions between the model protein apo-α-lactalbumin (apo-aLA) and the biosurfactant sophorolipid (SL) produced by the yeast *Starmerella bombicola*. SL occurs both as an acidic and a lactonic form; the lactonic form (lactSL) is sparingly soluble and has a lower critical micelle concentration (cmc) than the acidic form [non-acetylated acidic sophorolipid (acidSL)]. We show that acidSL affects apo-aLA in a similar way to the related glycolipid biosurfactant rhamnolipid (RL), with the important difference that RL is also active below the cmc in contrast to acidSL. Using isothermal titration calorimetry data, we show that acidSL has weak and saturable interactions with apo-aLA at low concentrations; due to the relatively low cmc of acidSL (which means that the monomer concentration is limited to ca. 0–1 mM SL), it is only possible to observe interactions with monomeric acidSL at high apo-aLA concentrations. However, the denaturation kinetics of apo-aLA in the presence of acidSL are consistent with a collaboration between monomeric and micellar surfactant species, similar to RL and non-ionic or zwitterionic surfactants. Inclusion of diacetylated lactonic sophorolipid (lactSL) as mixed micelles with acidSL lowers the cmc and this effectively reduces the rate of unfolding, emphasizing that SL like other biosurfactants is a gentle anionic surfactant. Our data highlight the potential of these biosurfactants for future use in the detergent and pharmaceutical industry.

## Introduction

Surfactants are surface-active agents which find use in a wide range of applications, including paints, emulsions, fabric softeners, detergents, and cosmetics (Otzen, 2011). They are often co-formulated with proteins, including peptides and enzymes, in a range of products such as detergents and pharmaceuticals. Surfactants often interact with proteins in such formulations, depending on parameters such as the charge of the surfactant and protein, pH and ionic strength. The interactions can be beneficial, leading to e.g., enhanced enzyme activity and reduced aggregation propensity, or harmful, usually by denaturing and inactivating proteins. This is a particular problem in the detergent industry where enzyme activity during washing processes is required. The influence of surfactants on protein stability and unfolding has been extensively reviewed by Otzen (2010, 2011, 2015).

Current surfactants used in industry are largely manufactured from petrochemical or oleochemical sources using synthetic chemistry (Ash and Ash, 2010). However, nature provides attractive alternatives. A growing number and range of microorganisms have been shown to produce different types of amphiphilic surface-tension reducing compounds called biosurfactants. The production processes (fermentation and purification) have been extensively investigated (Sen, 2010), and some biosurfactants are emerging as natural and sustainable alternatives to synthetic surfactants. The glycolipid class, in which different sugars are linked to linear or branched hydrophobic moieties usually derived from fatty acids, has been identified as one of the most promising alternatives to synthetic surfactant because of the high production yields through fermentation, with reported yields of several hundred g/L (Daniel et al., 1998).

**FIGURE 1:**
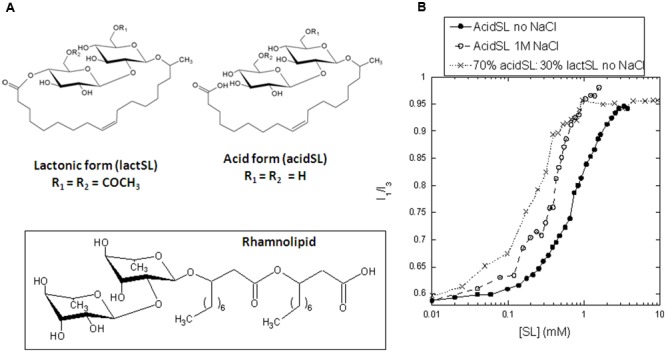
**(A)** Structure of acidic and lactonic sophorolipids used in this study. The structure of rhamnolipid is shown for comparison. **(B)** Determination of the critical micelle concentration of pure acidSL and a mixture of 70% acidSL and 30% lactSL using pyrene fluorescence.

In order to successfully replace synthetic surfactants with biosurfactants, it is important to understand how biosurfactants interact with proteins and how this affects protein stability and function compared with synthetic surfactants. As reviewed by Otzen (2016), relatively few studies have addressed biosurfactant–protein interactions. These studies have mainly focused on the class of biosurfactants known as glycolipids, in which mono- or di-saccharides of rhamnose or disaccharides of glucose [e.g., glucose-β-1,2-glucose (sophorose), glucose-β-1,4-glucose (cellobiose), and glucose-α-1,1-glucose (trehalose)] are combined with different hydrophobic moieties. The best studied class, that of the RL, has a hydrophobic moiety consisting of two fused fatty acids, leading to a branched alkyl chain with an ester bond as well as a free carboxyl group. The carboxylic group makes RL anionic above pH 5–6. In SL, the sophorose group is attached subterminally to the fatty acid, leading to a methylated alkyl chain that can either terminate in an ionizable carboxyl group (like rhamnolipids) or close intramolecularly to form a circular lactone (**Figure 1A**). Previous studies suggest that biosurfactants constitute a unique class of surfactants which combine features from both non-ionic and ionic surfactants. This in contrast to traditional petrochemical derived surfactants which are exclusively anionic or non-ionic. Like many other surfactants and hydrophobic compounds, RL binds to bovine serum albumin, but only as monomeric ligands; there was no evidence for cooperative binding accompanied by unfolding which occurs in the presence of denaturing surfactants such as sodium dodecyl sulfate (SDS; Sanchez et al., 2008). Overall, RL is a mild surfactant: it does not affect the stability of commercial enzymes such cellulases, phospholipases and lecitase and even stabilizes α-amylases (Madsen et al., 2015). It also allows refolding of the bacterial outer membrane protein A (OmpA) (Andersen and Otzen, 2014b). However, it can denature the model proteins apo-aLA and myoglobin (Mb), though by very different mechanisms (Andersen and Otzen, 2014a). Apo-aLA is unfolded by RL monomers below the cmc (around 0.1–1 mM) and Mb by RL micelles above the cmc. Yet, in both cases denaturation leads to a highly helical conformation and the binding of almost the same amount of RL per weight (1.1–1.3 g/g protein) as for SDS. Nevertheless, unfolding kinetics in RL are 2–3 orders of magnitude slower than in SDS and much less complex, involving only one exponential phase, just as reported for the synthetic non-ionic alkyl maltosides (Otzen et al., 2009). This remarkable “gentle anionic” nature undoubtedly stems from RL ’s complex structure, where the bulky and branched alkyl chain may prevent effective packing against proteins, unlike SDS’ straight alkyl chain, and the carboxylate group of RL is not as acidic as the SDS sulfate group.

Sophorolipid are synthesized intracellularly in the anionic form by joining a hydroxylated fatty acid and two glucose molecules, leaving the carboxyl group free. At this stage, acetylation of one or both glucose molecules is possible as well. Next, these acidic SL are transported to the extracellular environment. Here, an extracellular lactone esterase can additionally transform the SL (with a preference for the di-acetylated acidic SL form) to the non-ionic lactonic form (Ciesielska et al., 2016). Unless further modified, industrial SL is thus a mixture of anionic and non-ionic forms. The extent of acetylation and lactonization of SL produced by the wild-type yeast is to a certain extent influenced by fermentation conditions. Overall, it has been suggested that unfavorable conditions trigger accumulation of acidic structures (Davila et al., 1997). Utilization of vegetable oils having the wrong fatty acid profile (e.g., depleted of oleic acid, rich in polyunsaturated fatty acids) will result in an increased acid/lactone ratio (Davila et al., 1994). The same trend is observed when too much yeast extract is applied, or when oxygen transfer is suboptimal (Casas and Garcia-Ochoa, 1999).

This study reports on the interaction between sophorolipid and the protein apo-aLA whose interplay with different synthetic surfactants has been studied extensively (Otzen et al., 2009; Malmendal et al., 2010). The apo-form of aLA lacks a highly stabilizing Ca^2+^ ion, making it very sensitive to destabilizing conditions. As a consequence, the protein even unfolds in the presence of non-ionic surfactants such as alkyl maltosides which normally do not interact with globular proteins, and this makes apo-aLA a very sensitive probe for structural changes caused by mild denaturants such as RL as described above (Andersen and Otzen, 2014a). As main type of SL we use a non-acetylated 100% anionic form (acidSL), which is highly soluble in water. We have also purified a 100% non-ionic form of lactSL, but due to its very low solubility we have only been able to study its effect in mixed micelles with acidSL. AcidSL has a slightly higher cmc than RL. Although SL shows the same level of interaction with apo-aLA as RL does in terms of binding stoichiometry, denaturation only occurs at and above the cmc. Based on isothermal titration calorimetry (ITC), we demonstrate that interactions between monomeric acidSL and apo-aLA are so weak and saturable that they can only be driven by high apo-aLA concentrations, while the kinetics of denaturation are consistent with a collaboration between monomers and micelles in the denaturation process.

## Materials and Methods

### Materials

A natural SL mixture of acidic and lactonic molecules was obtained by cultivating the yeast *Starmerella bombicola* ATCC 22214 as described (Saerens et al., 2011). AcidSL, main molecular weight 622 Da (C18:1), and lactSL, main molecular weight 688 Da (C18:1), were prepared to high purity as described (Roelants et al., 2016). Mixtures of these products were combined as described below.

### Methods

All experiments were carried out in 20 mM sodium phosphate pH 7 and 5 mM EDTA to ensure that all Ca^2+^ is removed from aLA, leaving it in the apo-state. Unless otherwise stated, all experiments were performed as described (Andersen and Otzen, 2014a) and briefly summarized here. Pyrene fluorescence was used to determine the critical micelle concentration of the two forms of SL by incubating 1 μM pyrene with different concentrations of SL and recording fluorescence spectra with an excitation wavelength of 335 nm and emission from 360 to 410 nm to obtain the ratio of emission at 372.5 (*I*_1_) and 383.5 nm (*I*_3_). The Trp fluorescence of 2 μM apo-aLA at different SL concentrations was recorded on an LS-55 spectrophotometer (Perkin-Elmer Instruments, Wellesley, MA, USA) using excitation at 295 nm, scanning speed of 200 nm/min, spectral band widths of 10 nm and emission from 310 to 400 nm. Narrower slit widths unfortunately resulted in poor spectra. Near-UV CD spectra were recorded on a JASCO J-810 spectropolarimeter (Jasco Spectroscopic Co. Ltd., Japan) with a bandwidth of 2 nm, a scanning speed of 50 nm/min and a response of 2 s over 320–250 nm in a 1-cm cuvette using 1 mg/ml apo-aLA in different concentrations of SL. Six accumulations were averaged and buffer background contributions were subtracted. In ITC experiments performed on a VP-ITC calorimeter (MicroCal, Inc., Northampton, MA, USA)., 75 mM acidSL was titrated into solutions of 0–3.0 mg/ml aLA in steps of 5 μl (after an initial injection of 3 μl) with 300 s spacing of injections at 28°C.

Stopped-flow kinetics carried out on an SX18-MV microreaction analyzer (Applied Photophysics, Leatherhead, UK) involved mixing of 22 μM apo-aLA in a 1:10 ratio with SL. Due to the low solubility of lactSL, it was not possible to measure interactions between apo-aLA and pure lactSL. To make a solution with 30% lactSL and 70% acidSL, 30 mg lactSL and 70 mg acidSL were weighed out, dissolved in 96% ethanol, dried by vacuum concentration under centrifugation and dissolved to 22 mM in buffer. It was not possible to obtain a solution of lactSL:acidSL with higher proportions of lactSL. For experiments involving unfolding of apo-aLA in SDS at 0.5 M NaCl, apo-aLA in 1 M NaCl was mixed 1:1 with different concentrations of SDS to avoid slow precipitation of SDS in NaCl which occurs on the minute time scale.

## Results

### AcidSL Binds to Apo-aLA in Several Binding Steps

Apart from stopped-flow kinetics, all our analysis of the impact of SL on apo-aLA is confined to acidSL, since the low solubility of lactSL ruled out measurements with lactSL alone and limited experiments to lactSL:acidSL mixtures with 18–30% weight percent lactSL. We start out by determining the cmc of the acidSL preparation. The cmc is sensitive to solvent conditions such as pH and buffer strength (Jönsson et al., 1998) and needs to be determined for each specific experimental condition that is applied during investigations. In the well-established pyrene fluorescence assay, the characteristic *I*_3_/*I*_1_ ratio starts to rise around 0.3 mM acidSL and levels off around 3 mM (**Figure 1B**). These values are shifted twofold to threefold upward compared to RL, which under the same buffer conditions undergoes a pyrene transition between 0.1 and 1 mM RL (Andersen and Otzen, 2014a). We conclude that the cmc lies between 0.3 and 3 mM acidSL, depending on how one chooses to define it. The change in the *I*_3_/*I*_1_ ratio is midway around 0.8 mM. This is consistent with a cmc of 245 mg/ml or ∼0.4 mM recently determined in water by changes in surface tension (Roelants et al., 2016).

**FIGURE 2:**
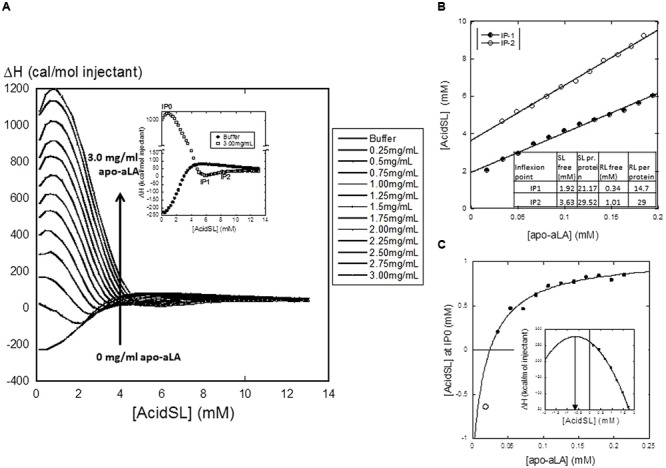
**(A)** Isothermal Titration Calorimetry (ITC) titration of acidSL into apo-aLA, showing the enthalpograms resulting from titration into different concentrations of apo-aLA. Each data point represents the total enthalpy for a single injection event. The insert defines the characteristic points IP0-IP2. **(B)** Plot of IP1 and IP2 versus protein concentration. Results from the linear fits are provided in the insert Table. This yields the stoichiometry (number of acidSL molecules per apo-aLA molecule) as well as the free concentration of acidSL at the two characteristic points. **(C)** Plot of IP0 versus protein concentration fitted to a binding isotherm with floating base line using the data points indicated by the filled circles (Eq. 2). This intercepts the *x*-axis at 25 μM apo-aLA and predicts an apparent dissociation constant of 20 ± 16 μM apo-aLA, an amplitude of 2.4 ± mM AcidSL and an intercept of the *y*-axis at -1.3 ± 1.2 mM AcidSL. The empty circle shows the negative peak position predicted for the ITC data obtained at 0.25 mg/ml apo-aLA (obtained from the parabolic fit shown in the insert). This point agrees well with the value predicted from the hyperbolic fit.

The next step was to determine how well acidSL binds to apo-aLA. We used the well-established ITC approach, wherein acidSL is titrated into a solution of apo-aLA and the associated heat change is recorded. The ensuing enthalpogram reveals different binding steps (**Figure 2A**). The salient features include three characteristic points which we designate IP0–IP2 (**Figure 2A**). IP0 is a highly exothermic step peaking at 0.7–0.8 mM acidSL irrespective of the protein concentration. The signal then declines to very close to zero with a minimum at IP1, followed by a small increase to a plateau level that is very close to that of buffer; the midpoint for this transition is IP2. The appearance of the enthalpogram is very similar to that of RL, except that RL shows a monotonic decline in enthalpy at low RL concentrations and does not have a distinct IP0 maximum (Andersen and Otzen, 2014a). According to the standard interpretation of these enthalpograms, a plot of IP versus protein concentration can be fitted to a linear equation of the following form (Nielsen et al., 2005):

(1) $$\begin{array}{c} {\left[\mathrm{Surf}\right]}_{\mathrm{total}}={\left[\mathrm{Surf}\right]}_{\mathrm{free}}+N_{\mathrm{surf}}\;*[\mathrm{protein}] \end{array}$$

This equation corresponds to a mass balance equation where [Surf]_free_ is the concentration of non-bound surfactant and *N*_free_ is the number of surfactant molecules bound per protein molecule. Our data yield an excellent fit for both IP1 and IP2 (**Figure 2B**) and the values (**Figure 2B**) are generally comparable to those of RL (also shown in the insert), except that the concentration of free SL at both characteristic points is somewhat higher. The concentration of free surfactant at the highest characteristic point (IP2) is usually taken as its cmc value, since that corresponds to the end of the binding process where all additional surfactant must form bulk micelles. This value is predicted to be 3.6 mM for SL, which is in the top range of the cmc value of 0.3–3 mM predicted from **Figure 1B**. The binding ratios of 21.2 and 29.5 SL per protein at IP1 and IP2 correspond to 0.93 and 1.29 g SL per protein. For IP0, the situation is different: the peak position shifts with protein concentration but in a hyperbolic rather than linear manner (**Figure 2C**). This obviously cannot be interpreted within the confines of the simple mass-conservation balance described by Eq. 1. Instead we fit an equation describing a simple saturable binding isotherm with a floating end level to these data:

(2) $$\begin{array}{c} {}[\mathrm{AcidSL}]={\mathrm{Amplitude}}^{*}[apo-aLA]/([apo-aLA]+K_{\mathrm{diss}})+\mathrm{offset} \end{array}$$

The fit is excellent provided it intercepts the *x*-axis at 25 μM, leading to an estimated dissociation constant of 20 ± 1 μM apo-aLA. We return to the significance of this curve in the section “Discussion.”

### SL Unfolds Apo-aLA Around the cmc of AcidSL

**FIGURE 3:**
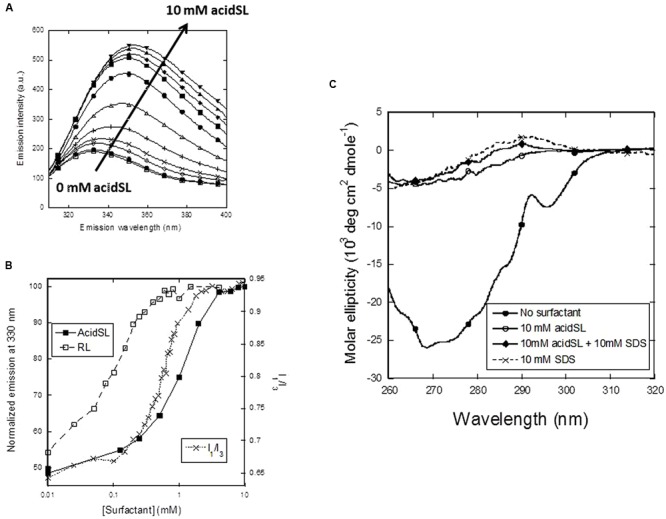
**Unfolding of apo-aLA in acidSL. (A)** Trp fluorescence emission spectra undergo a red shift at higher acidSL concentrations. **(B)** Trp fluorescence plots of apo-aLA in acidSl and RL (left axis) compared with the pyrene plot of apo-aLA in SL (right axis). **(C)** Near-UV CD spectra of apo-aLA in different surfactants.

To monitor the consequences of these changes at the structural level, we first turn to Trp fluorescence, which is a very sensitive probe to follow conformational changes as a function of surfactant concentration. Between 0 and 10 mM, AcidSL leads to a marked red-shift in the fluorescence spectrum of apo-aLA (**Figure 3A**) which follows the change in pyrene emission quite well (**Figure 3B**), indicating that the conformational changes are linked to formation of micelles. In contrast, RL induces a change in fluorescence at a much lower concentration. We confirmed that the fluorescence change is linked to the loss of tertiary structure using near-UV circular dichroism, which is a very sensitive determinant of the amount of tertiary structure. Native apo-aLA has a very characteristic near-UV spectrum (**Figure 3C**), with a large minimum around 270 nm due to the immobilization of Trp residues in the tertiary structure of apo-aLA. However, 10 mM acidSL completely removes this characteristic signature, leading to the same low level of structure caused by 10 mM SDS (**Figure 3C**). The large absorbance of acidSL at low wavelengths precluded the use of far-UV CD to probe changes in secondary structure.

### Kinetics of Unfolding of Apo-aLA in AcidSL and the Effect of LactSL

**FIGURE 4:**
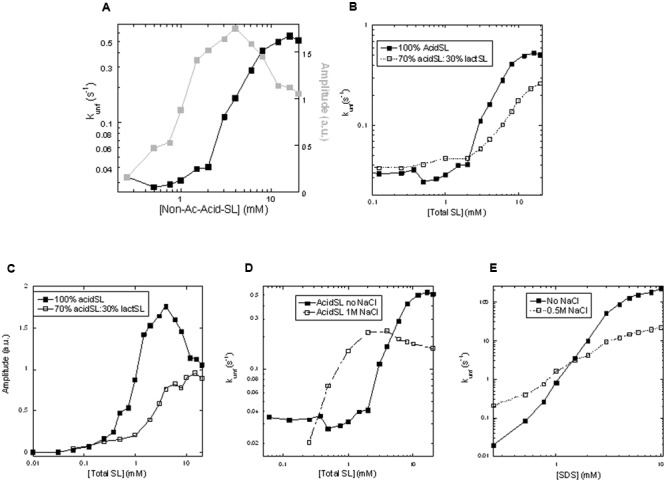
**Kinetics of unfolding of apo-aLA in SL. (A)** Amplitudes and rate constants in pure acidSL. **(B)** Rate constants and **(C)** amplitudes of unfolding in pure acidSL and mixtures of 70% acidSL and 30% lactSL. **(D)** Rate constants of unfolding of apo-aLA in acidSL in 0 and 1 M NaCl. **(E)** Rate constants of unfolding of apo-aLA in sodium dodecyl sulfate (SDS) in 0 and 1 M NaCl.

Our previous measurements have concentrated on binding interactions under equilibrium conditions. To obtain more insight into the steps involved in binding and denaturation, we now turn to the kinetics of the associated conformational changes. This is accomplished by stopped-flow kinetics, where apo-aLA is rapidly mixed with different concentrations of SL and the changes in fluorescence are followed. In all cases, a single exponential decay with associated amplitude and rate constant is observed. Between 0 and 20 mM acidSL, the amplitude rises more steeply at low acidSL concentrations than the rate constant (**Figure 4A**), just as was observed for RL (Andersen and Otzen, 2014a). Unfolding signals start to appear around 0.3 mM which is in the region where micelles start to form, confirming the need for acidSL micelles to unfold apo-aLA. The unfolding amplitude closely follows the fluorescence signal increase under equilibrium conditions (**Figure 3B**) up to around 4 mM acidSL, after which the amplitude declines while the equilibrium reaches a plateau. This decline may be due to burst-phase binding of SL to apo-aLA in the dead-time of mixing before the actual unfolding occurs, which will restrict the amount of signal change that can occur. Unfortunately further speculation on this topic is challenged by the fact that the sophorolipid itself contributes significantly to the overall level of the fluorescence signal (which integrates all emission above 320 nm) and therefore contributes substantially to the signal change during the dead-time of mixing.

We then turned to mixed micelles containing 30% by weight of lactSL in the SL mixture. Inclusion of the non-charged lactSL reduced the cmc considerably (**Figure 1B**); pyrene values start to rise around 0.03 mM SL and level off around 0.8 mM. Consistent with this, a direct measurement of the cmc of lactSL (using Total Organic Carbon analysis to measure the low solubility of this compound) found the cmc to be around 0.07 mM (Roelants et al., 2016). Despite this reduction in cmc (and the concomitant availability of micelles at even lower concentrations than for pure acidSL), we observed a ca. 50% reduction in the unfolding rate compared to acidSL above the cmc, though rates below the cmc were comparable to those of acidSL (**Figure 4B**). There is also a smaller and monotonic rise in the amplitude (**Figure 4C**) without any subsequent decline, suggesting that there was no significant binding of the mixed acidSL-lactSL micelles to apo-aLA prior to unfolding. Thus inclusion of the more hydrophobic lactSL does have an impact on the unfolding kinetics of apo-aLA. This suggests that the electrostatic interactions mediated by the carboxylic acid group on acidSL (which is absent in lactSL) are important to stabilize contacts with the protein. We confirmed the importance of these interactions by repeating the experiment with inclusion of 1 M NaCl. High ionic strength will reduce the strength of electrostatic interactions due to the increase in the dielectric constant. They also decrease the cmc of ionic surfactants for the same reason, and 1 M NaCl indeed results in a decrease of the cmc by around a factor of 2 (**Figure 1B**). Indeed, in 1 M NaCl rates of unfolding are decreased by a factor 3 at high acidSL concentrations (**Figure 4D**) compared to salt-free conditions. At lower acidSL concentrations (∼0.5–4 mM), the unfolding rates are somewhat increased by NaCl. The same limited enhancement of rates around and slightly above the cmc is seen when apo-aLA is unfolded in SDS (**Figure 4E**). This low-concentration effect (also seen for the mixed acid-lactSL in **Figure 4B**) may be explained by the lower cmc values which means that there will be a higher micelle concentration at these low concentrations.

## Discussion

### SL Is Even Gentler as Protein Denaturant than RL

In this study, we have analyzed how the biosurfactant sophorolipid interacts with the surfactant-sensitive model protein apo-aLA, using both pure acidSL and mixtures of acidic SL and acetylated lactonic SL. Here, we discuss our results also in light of results obtained with rhamnolipid from a previous report (Andersen and Otzen, 2014a). We start by comparing acidSL with RL, given that they are both anionic at neutral pH thanks to a free carboxyl group. Their hydrophobic moieties differ in that RL has a branched alkyl chain with two C_7_ chains (in total 18 CH/CH_2_/CH_3_ atoms) and an internal ester bond while acidSL also has 18 CH/CH_2_/CH_3_ atoms, but a double bond rather than an ester bond. Their colloidal properties only differ slightly. RL has a twofold to threefold lower cmc, which indicates that it is more favorable to pack its hydrophobic moiety into a micelle structure than that of acidSL. RL also reduces surface tension to as little as 26 mN/m (admittedly in 0.1 M NaHCO_3_, used to maintain an alkaline pH and keep RL ionized) (Hisatsuka et al., 1971), while lactSL only reduces it to around 40 mN/m in water (Roelants et al., 2016). This is further support of RL ’s superior packing abilities, since a reduction in surface tension reflects the ability to pack well at the air–water interface. Superior packing reports on intermolecular surfactant interactions, but this does not *a priori* predict that RL should be better at binding to proteins. Indeed, SL shows a slightly higher level of binding to apo-aLA at the first characteristic point (21 versus 15 surfactant molecules per apo-aLA molecule), though both surfactants converge to around 29–30 molecules at saturation, and both lead to complete loss of tertiary structure by near-UV CD. However, it turns out that RL is slightly more aggressive toward apo-aLA than acidSL is, starting the denaturation process around 0.025 mM RL, which is well below its cmc, while acidSL only really starts the denaturation process in parallel with the accumulation of micelles and reaches a plateau at 4 mM, which is 1–2 mM after the pyrene fluorescence ratio has leveled out (**Figure 3B**). This indicates that monomeric RL is able to denature apo-aLA whereas acidSL has to include micelles to achieve this. Nevertheless, the postulated difference hinges on a small number of data points and it should be noted that complete denaturation of apo-aLA only occurs around 0.5 mM RL where a substantial population of micelles has built up, so it may rather be a question of monomeric RL playing a more active role together with increasing concentrations of micelles.

### Evidence for Weak and Saturable Interactions between SL and Apo-aLA at Low SL Concentrations

We also interpret our data to mean that the high concentration of free SL predicted from the ITC measurements (2–4 mM at the two characteristic points) may be in the form of micellar acidSL in both cases, rather than monomeric acidSL. If monomeric acidSL does not denature apo-aLA, it is more likely that the interactions around IP1 and IP2 only involve micellar acidSL which probably interacts rather weakly with the protein. This is satisfactorily corroborated by the interaction marked by the peak at characteristic point IP0. The hyperbolic curve in **Figure 2C** is a remarkable deviation from the customary linear fits that one obtains from enthalpograms which monitor interactions between proteins and strongly binding anionic surfactants like SDS (Nielsen et al., 2005). These linear fits are a consequence of a simple mass-balance between bound and free surfactant which assumes in essence almost a phase separation between a reservoir of free surfactant and surfactant bound to protein, rather than a saturable equilibrium between bound and free ligand (surfactant). This is analogous to the “hard” titration used, e.g., to measure protein-ligand stoichiometries by adding increasing amounts of ligand to a certain concentration of protein to form a complex with a very low dissociation constant (much lower than the protein concentration); under these conditions all added ligand will form a complex with the protein right up to a break point where all protein binding sites are filled up, after which complex formation abruptly stops and all subsequently added ligands remain unbound (Mogensen et al., 2002). This break point will shift upward with increasing protein concentration in a proportional fashion, although there will not be a reservoir of free ligands in “phase equilibrium” with complexed ligands. The curve illustrates two important findings: firstly, interactions at these low acidSL concentrations are governed by weak and saturable interactions between protein and surfactant, rather than hard “all-or-none” binding. Secondly, these interactions only manifest themselves at a minimum concentration above ∼25 μM (0.375 mg/ml) apo-aLA. The hyperbolic curve formally predicts that at concentrations below this threshold value, the endothermic peak position occurs at negative acidSL concentrations, i.e., there is no actual peak at these low concentrations. This is consistent with measurements at 0.25 mg/ml apo-aLA where the peak is conspicuously absent and is predicted to occur at values <0 mM acidSL (**Figure 2C**). In other words, the interactions reported by the peak at IP0 must involve monomeric acidSL. Accordingly, they cannot occur to any significant extent at low apo-aLA concentrations because the protein concentration is too low to drive binding, and this cannot be compensated by increasing the acidSL concentration since this will eventually (above 0.1 mM acidSL) lead to formation of micelles which interact with apo-aLA in a different manner, i.e., according to IP1 and IP2. Only if the protein concentration increases so much that it exceeds the actual dissociation constant of the reaction will it be possible to get strong interactions between monomeric acidSL and apo-aLA. A similar type of interaction may be occurring with RL, but the data are less unequivocal on this point since there is no peak in the IP0 regime, but only a monotonic decrease in the enthalpy of injection (Andersen and Otzen, 2014a). Building on our insight from the hyperbolic regime in **Figure 2C**, it may be argued that this implies that all the peaks are (formally) found at negative RL concentrations (like the value measured for acidSL with 0.25 mg/ml apo-aLA). That is, they will not occur at higher apo-aLA concentrations because the IP0 interaction is actually weaker for RL or is more effectively competed out by the micellar interactions which take over as we get close to and above the cmc.

### Unfolding Kinetics Involve Collaboration between Monomeric and Micellar Species

As a consequence of the threshold concentration of apo-aLA required to obtain IP0 transitions, the interactions monitored by steady-state fluorescence and stopped-flow kinetics, which all take place at around 2 μM apo-aLA, only report on interactions between micellar acidSL and apo-aLA. Accordingly, we observe two regimes of interactions: firstly, one around 0.2–2 mM acidSL (corresponding to the concentration range leading up to IP1 where there is up to 2 mM free acidSL) with a low and relatively constant unfolding rate constant but a steeply increasing amplitude which stabilizes around 2 mM acidSL. In this region there is a build-up of both monomeric acidSL and micelles which reaches saturation around 2 mM acidSL according to our pyrene titration. Secondly, a regime from 2 mM to the end of the measured region (20 mM), where the micelle concentration continues to rise, leading to an increase in the rate constant but a constant and eventually declining amplitude. We have reported a similar behavior for apo-aLA in the presence of non-ionic surfactants like alkyl maltosides and zwitterionic phosphocholines (Otzen et al., 2009), in which the rate constant only starts to rise around and above the cmc, but reaches a plateau level which increases proportional to the cmc of the surfactants involved. We interpreted this to mean that denaturation by these types of surfactants involves a collaboration between monomeric and micellar species to unfold the protein (Otzen et al., 2009). In other words, monomers are necessary to facilitate unfolding but the proportion of unfolded protein (amplitude) increases in the concentration range where the micelle concentration builds up in parallel with monomer concentration, after which the unfolding rate constant increases proportional to the micelle concentration until a certain plateau level has been reached. This differs radically from the behavior of strong anionic surfactants like SDS which largely reach their peak unfolding rate constants below and up to the cmc (Otzen et al., 2009). AcidSL sides squarely with the non-ionic/zwitterionic surfactants in this regard, emphasizing its nature as a relatively mild surfactant in much the same way as RL.

### Inclusion of lactSL in Mixed Micelles Emphasizes the Role of the cmc in Denaturation

The role of the monomeric species of surfactant in denaturing apo-aLA is borne out by our data with lactSL. Inclusion of lactSL together with acidSL leads to a lowering of the overall cmc in agreement with previous reports (Roelants et al., 2016), and this in turns means that there is less monomer present to help the denaturation process. As a consequence, the unfolding rate constant does not increase to the same level as in acid SL, reaching a plateau level of around 0.3 s^-1^ in mixed acidSL/lactSL micelles rather than 0.5 s^-1^ in pure acidSL (**Figure 4B**). Rate constants of unfolding in mixed micelles are slightly higher around the cmc region, because the lowered cmc means that the micelle concentration is slightly higher in this region and that can to some extent compensate for the lowered monomer concentration – until we reach the higher micelle concentrations (above 2–3 mM SL) where there is less monomer to “help out” the mixed micelles than the pure acidSL micelles. This behavior is corroborated by another approach to lower the cmc, namely to use high concentrations of NaCl. In this case, rate constants increase markedly at low SL concentrations because of the increase in micelle concentration but again end up at a lower plateau level.

In summary, we have shown that sophorolipids can be used as gentle surfactants which act by a combination of monomeric and micellar species to denature apo-aLA. Monomeric acidSL is not able to denature apo-aLA in the low μM protein range, because the weak binding and saturable binding reactions either require large concentrations of apo-aLA or are superseded by micelle formation at higher concentrations. The use of lactonic sophorolipids lowers the cmc further, thus effectively decreasing the denaturing potency at the same time as it lowers surface tension more effectively than acidSL. This augurs well for the use of sophorolipids as alternatives to chemical surfactants in the detergent industry.

## Author Contributions

KA and DO planned the experimental design. KA, BV, and DO carried out the experiments. DO, IB, and SR supervised the experiments. DO wrote the manuscript with input from KA, IB, and SR.

## Conflict of Interest Statement

The authors declare that the research was conducted in the absence of any commercial or financial relationships that could be construed as a potential conflict of interest.

## Abbreviations

AcidSL: non-acetylated acidic sophorolipid
apo-aLA: apo-α-lactalbumin
lactSL: di-acetylated lactonic sophorolipid
cmc: critical micelle concentration
RL: rhamnolipid
SL: sophorolipid
